# Patient-Perceived Satisfaction and Knowledge Uptake in a Combined Cardio-Obstetrics Clinic

**DOI:** 10.3390/jcdd9120433

**Published:** 2022-12-02

**Authors:** Karen L. Florio, Darcy White, Kensey Gosch, Neil Patel, Tara Daming, Emily M. Williams, Sarah Hostetter, Rebecca Gray, Lynne Nelson, Kathleen Swearingen, Christine Henricks, Anna Grodzinsky, Valerie Rader, John Lee, Anthony Magalski, Laura Schmidt

**Affiliations:** 1 Women’s and Children’s Division, Saint Luke’s Hospital of Kansas City, 4401 Wornall Road, Kansas City, MO 64111, USA; 2 Department of Obstetrics and Gynecology, University of Missouri-Kansas City School of Medicine, 5000 Holmes Street, Kansas City, MO 64110, USA; 3 Department of Obstetrics and Gynecology, University of Missouri School of Medicine, 1 Hospital Drive, Columbia, MO 65212, USA; 4 Department of Cardiology, Mid-America Heart Institute, Saint Luke’s Hospital of Kansas City, 4401 Wornall Road, Kansas City, MO 64111, USA; 5 Department of Obstetrics and Gynecology, University of Kentucky School of Medicine, 800 Rose Street, Lexington, KY 40536, USA; 6 Department of Obstetrics and Gynecology, Mercy Hospital of Saint Louis, 621 S New Ballas Road, Saint Louis, MO 63141, USA; 7 Department of Obstetrics and Gynecology, University Health Truman Medical Center, 2301 Holmes St, Kansas City, MO 64108, USA; 8 Department of Maternal-Fetal Medicine, Mercy Hospital of Springfield, 1235 E Cherokee Street, Springfield, MO 65804, USA; 9 Department of Cardiology, University of Missouri-Kansas City School of Medicine, 5000 Holmes Street, Kansas City, MO 64110, USA

**Keywords:** cardio-obstetrics, heart disease, pregnancy, maternal mortality, patient satisfaction

## Abstract

Heart disease is the leading cause of pregnancy-related mortality in the United States and has led to the development of combined cardio-obstetrics (COB) clinics as a model for prenatal care. In other areas of medicine, these types of collaborative care models have shown improvement in morbidity, mortality, and patient satisfaction. There is some data to suggest that a combined COB clinic improves maternal outcomes but there is no data to suggest patients prefer this type of care model. This study aims to evaluate patient satisfaction in a combined COB clinic and whether this type of model enhances perceived communication and knowledge uptake. A quality questionnaire was developed to assess patient perceptions regarding communication, satisfaction, and perceived knowledge. Patients who attended the clinic (*n* = 960) from 2014–2020 were contacted by email, with a response received from 119 (12.5%). Participants completed a questionnaire assessing satisfaction and perceived knowledge uptake with answers based on a Likert scale (7 representing very satisfied and 1 representing very unsatisfied). Safe and effective contraceptive use was evaluated by multiple choice options. Knowledge was also assessed by comparing contraceptive use before and after the clinic. Participants reported high levels of satisfaction with the clinic (6.2 ± 1.5), provider-to-patient communication (6.1 ± 1.6), and with the multidisciplinary appointment approach (6.3 ± 1.5). As well, participants reported an increase in knowledge about heart disease a result of collaborative counseling. In summary, a multidisciplinary approach to cardio-obstetrics not only improves outcomes but is a patient satisfier.

## 1. Introduction

The United States maternal mortality rate ranks amongst the highest in the industrialized world and continues to rise at an alarming rate [1,2]. Childbirth is now the 9th leading cause of mortality in women aged 20–44 [3]. Through coordinated efforts of state-run maternal mortality review committees, cardiovascular disease (CVD) has been recognized as the leading cause of pregnancy-related death [4]. One of the challenges facing medical professionals is how to best manage this diverse group of patients. Traditional medical teaching dictates that each organ system is managed by different medical specialists, which leads to siloed and disjointed care. Recently, the American Heart Association released guidance regarding the importance of a multidisciplinary approach in caring for these high-risk patients in order to optimize pregnancy outcomes [5,6]. However, they were not prescriptive in their recommendations, leaving room for innovation and newer care models to fill that void.

The Heart Disease in Pregnancy Program (HDPP or COB) of the Saint Luke’s Hospital of Kansas City, an affiliate of the University of Missouri-Kansas City School of Medicine (UMKC), was developed in 2014 to address the growing population of CVD in pregnancy. This novel clinical care model provides an individually tailored medical approach to each patient, with a cardiologist, maternal-fetal medicine specialist, nurse care coordinator, and often, an anesthesiologist all present during the same visit. This type of care model has led to better outcomes including less use of intensive care resources, less cesarean sections, higher birth weights and higher gestational ages at delivery [7].

Pregnancy can be a time of significant vulnerability and having multiple providers in an exam room may be an intimidating situation. This can lead to reluctance towards honest communication or hesitancy to disclose important medical history, including the desire for specific contraception or even termination. However, a multidisciplinary approach to clinical care has been shown to improve patients’ quality of life, understanding of disease processes and overall satisfaction [8,9]. Higher patient satisfaction scores often correlate with improved clinical outcomes [10]. Studies have shown that clear communication amongst the patient care team, respectful delivery of that care, and simplified explanations of the treatment plan are all patient satisfiers, which are improved through multidisciplinary care delivery [11,12]. As cardio-obstetrics is a relatively new specialty, there are no data published indicating patients prefer this type of model to traditional prenatal care [13]. The purpose of this study was to assess patient satisfaction and perceived knowledge gained through a multidisciplinary care model in the cardio-obstetrics program at Saint Luke’s Hospital of UMKC.

## 2. Materials and Methods

The Saint Luke’s Hospital cardio-obstetrics clinic is a multidisciplinary program housed in the maternal-fetal medicine unit and operates one half day per week. Birthing persons with heart disease (known or suspected congenital, acquired, or arrhythmic) from anywhere across a 4-state catchment area are evaluated by a cardiologist and maternal-fetal medicine provider simultaneously at each visit, accompanied by a dedicated nurse coordinator and, intermittently, nurse practitioners and cardiology and/or maternal-fetal medicine fellows. A thorough history and physical exam is performed at each visit and discussions surrounding pregnancy physiology and the impact these changes pose on individualized disease states are reviewed. A standardized form created by the cardio-obstetrics team outlining recommendations for antenatal care, mode, timing and location of delivery, postpartum follow up, and frequency of both maternal and antenatal imaging is delineated and documented in the electronic medical record. Safe and effective contraception is discussed and offered, generally at the third trimester or postpartum visit. The majority of patients evaluated receive a one-time consultation and continue routine obstetrical care at their local institution. Approximately 20% of those assessed transfer to maternal-fetal medicine for the remainder of their prenatal care and delivery at Saint Luke’s, a level III maternity care center, due to the high risk nature of their cardiac condition. Patients can present to the cardio-obstetrics clinic at any time during gestation and therefore the number of combined visits varies for each individual. In addition to weekly clinical visits, the multidisciplinary team consisting of cardiology, maternal-fetal medicine, obstetrics, anesthesia, a cardio-obstetrics nurse coordinator, obstetrics and ICU nursing, cardiology and maternal-fetal medicine fellows, and occasionally, cardiovascular surgery, meet quarterly to discuss patient care and opportunities for improvement (debriefings). A detailed list of birthing persons including name, age and type of CV disease is kept by the nurse coordinator in the MFM office.

Birthing persons aged 18 years and older who attended the Saint Luke’s Hospital cardio-obstetrics clinic from 2014–2020 were contacted by the marketing department via email and asked to participate in a short questionnaire to assess their satisfaction with their experience in the clinic. Contact information was obtained from the electronic medical record and for those unreachable through email, an additional attempt was made through the Saint Luke’s patient portal. The questionnaire was previously tested on a random sampling of patients delivered via phone conversation by an OB/GYN resident to assess applicability. For this study, attempts through the phone were not made as it was felt this would be too time consuming given the hospital-wide staff shortages during COVID. Pregnant persons at any gestational age with various types of cardiac symptoms (including but not limited to shortness of breath, syncope, palpitations, chest pain) or known underlying cardiac conditions (i.e., genetic or acquired arrhythmias, congenital cardiac anomalies or acquired cardiovascular disease such as cardiomyopathy) evaluated in the clinic during the aforementioned time frame were included. Overall satisfaction and perceived knowledge gained as a result of attending the clinic were gauged through a 37 question poll. The questions were programmed into a secure database (REDCAP) and an email with the REDcap link was sent to each participant for completion. Questions were iteratively developed by the clinical staff in order to assess the quality of care delivered, perceived communication, and knowledge uptake following at least one visit. Two attempts were made to contact patients and if after the second point of contact the email was not opened, this was considered a non-responder. Informed consent was previously obtained which included permission for any future contact. The study was approved through the Saint Luke’s Hospital of Kansas City IRB (#SLHS-21-009) as a quality improvement initiative.

### 2.1. Patient Satisfaction

Patients were asked to rate their satisfaction with various components of the clinic including the degree of communication between staff members, satisfaction with a combined visit, and the perceived quality of care received. Answers were based on a Likert scale, with 7 representing very satisfied, 6 representing satisfied, 5 representing somewhat satisfied, 4 representing somewhat unsatisfied, 3 representing unsatisfied, 2 representing very unsatisfied and 1 representing the worst medical encounter experienced. Option 0 was available for those who did not feel comfortable answering a question or for those questions that were considered not applicable. Patients were also asked about utilizing these services for a future pregnancy (answered as either “yes” or “no”) and how they were informed about the program prior to their appointment (through their cardiologist, family practice or primary care provider, general obstetrician).

### 2.2. Patient Perceived Knowledge

Questions assessing patient knowledge prior to enrolling in the clinic were distributed to compare to the quality of counseling provided during the combined visit. Specifically, questions on whether participants saw a cardiologist and/or a high-risk pregnancy provider prior to the combined visit and if that counseling was comparable to that received in the cardio-obstetrics clinic. Contraception and planned conception are an important aspect of counseling in the COB clinic and in an effort to assess the quality of counseling provided during each visit, questions evaluating prior contraceptive use and post-clinic knowledge were also developed. The answers were categorized into barrier, hormonal, and long-acting contraceptive methods (Appendix A). If multiple methods were used or if the specific type(s) of contraception could not be recalled, participants were instructed to mark combined.

Statistical analysis was performed in SAS 9.4. For descriptive statistics, continuous variables were reported as means and standard deviations. Categorical variables were reported as counts and frequencies. Differences in patient satisfaction between subgroups (race, education, marital status and insurance status) were tested using the Kruskal Wallice (KW), due to the ordinal nature of the Likert scale. All tests for statistical significance were 2-tailed and were evaluated at a significance level of 0.05. Knowledge questions were analyzed as yes or no responses and reported in frequencies. Types of birth control are reported as nominal data in percentages.

## 3. Results

### 3.1. Clinical and Demographic Characteristics

During the study timeframe, a total of approximately 1500 patients were evaluated in the Heart Disease in Pregnancy Program at Saint Luke’s Hospital. Of these, 960 patients had provided email addresses or had access to the patient portal of which 119 responded to the survey (12.4% response rate). On average, women who participated were white (84.9%), in their early 30s (30.9 ± 7.5), married (74.6%) and were college educated (33.9%) with private insurance (76.1%) (Table 1), which is similar to the demographics reported for maternity survey responders [14]. Nearly a quarter of participants (24.3%) had more than one pregnancy as part of the combined clinic. Cardiac disease was classified into 4 groups with 26.9% of those who responded having congenital (which included valvular) disease, 24.4% with various forms of arrhythmias (including Wolf-Parkinson White, supraventricular tachycardia, bradycardia and inappropriate sinus tachycardia), 15.1% with acquired (which included both ischemic and cardiomyopathy), and 33.6% classified as other (including unexplained syncope, palpitations, aortopathies and postural tachycardia syndrome). As a surrogate for social support, participants were asked if they had a significant other who was involved with their pregnancy and 89.9% responded yes. However, only 54.6% had a social support person attend their cardio-obstetrics visit. Of those who responded yes to having someone present, 95.2% believed having someone attend those visits with them was helpful to their understanding. To further gauge socioeconomic barriers to care, participants were asked about their ability to afford medications by indicating whether or not they ever had to choose between purchasing their cardiac medications and rent (10.3% answered yes to foregoing cardiac prescriptions). Similarly, participants were asked about having to decline their medications in order to buy groceries with 9.4% answering yes. Finally, participants were asked to mark “yes” if they ever had to choose to sacrifice clinical care due to transportation issues (4.2%), co-pays (2.5%), or lack of childcare (3.4%). Participants were also asked about their fears of the medical system including fear of discrimination as part of receiving healthcare (1.7%), fear of recommendation to terminate the pregnancy (0%), or fear of being made to feel bad about the decision to continue pregnancy (0%). None of the participants responded that they did not attend because they did not believe it would make a difference in their overall outcome and 3.4% stated they did not attend because they had significant life stressors preventing them from accessing care.

### 3.2. Contraceptive Use

Participants were polled on their use of contraception prior to the pregnancy and were instructed to mark all that apply for those utilizing multiple modalities. Prior to the pregnancy and participating in the program, 36.1% of women with cardiac disease were not using any contraception. Those who were utilizing contraception to prevent pregnancy, 14.3% indicated they had an intrauterine device, 2.4 % were using some form of long-acting systemic progesterone (Nexplanon or Depo-Provera), 13.4% were on progesterone-only oral pills, 14.3% were utilizing combined oral contraception, 15.1% were using condoms, 3.4% had a hormonal vaginal ring, 10.9% reported natural family planning, 10.1% endorsed some other form of contraception (spermicides, withdrawal methods or other not listed), and only one person reported using a contraceptive patch (0.8%) (Table 2).

### 3.3. Referral Patterns and Clinical Care Preferences

Forty eight percent of patients who participated in the survey reported having been counseled or were currently receiving prenatal care under the guidance of a high-risk obstetrician (maternal-fetal medicine [MFM]) prior to their consult in the combined cardio-obstetrics clinic (Table 3). Seventy two percent were evaluated by a cardiologist prior to their cardio-obstetrics visit. When asked about preferences for a combined visit, only 7.6% desired to have separate cardiology and obstetrics visits. When asked if a combined approach was intimidating, 14.3% responded that having both an obstetrician and a cardiologist in one room at the same time was indeed intimidating. However, 86.4% of women found this platform more conducive to enhanced communication between providers and 86.6% of respondents believed this combined model improved their overall pregnancy care. As well, 73.1% felt this type of care model saved them money throughout the course of the pregnancy. Eighty four percent of women attended their postpartum visit, either in the COB clinic or with their general OB provider. The majority of women (70.6%) interviewed would use the program for a subsequent pregnancy and would refer friends of family to the program (89.1%).

### 3.4. Patient Satisfaction

Overall patient satisfaction with the combined cardio-obstetrics clinic was high (Table 4). On a 7-point scale with 1 being low satisfaction and 7 being the highest satisfaction, the mean score for perceived quality of care received was 6.2 ± 1.5. The mean satisfaction score for seeing both a maternal-fetal medicine provider and cardiologist within the same visit was 6.3 ± 1.5. Patients were overall satisfied with both communication amongst providers (6.1 ± 1.6) and communication with the patient herself about her disease process and expectations (6.1 ± 1.5). Many patients evaluated in the COB clinic return to their community OB providers for delivery but for those requiring a higher level of care, they are transferred to MFM for delivery at the tertiary care center. For those women who delivered at the main hospital campus with MFM, the communication perceived between the clinic providers and the delivery team was high (6.0 ± 1.7). Patients delivered at their community hospitals perceived even higher communication between the COB clinic providers and their delivery team (6.1 ± 1.6).

### 3.5. Perceived Knowledge Gained

Women were asked about their educational counseling prior to becoming a patient in the combined COB clinic (Table 5). Less than half of participants (35%) were counseled by their cardiologist about the dangers a pregnancy may pose on specific heart conditions. Only 30.5% were previously counseled about the risks and benefits of their current medication regimen during a future pregnancy. Eleven percent of participants were counseled against a future pregnancy altogether prior to becoming a patient in the combined COB clinic. After being counseled in the COB clinic, 73.9% of patients reported a gain in knowledge about heart disease and the risks pregnancy pose on disease, but only 55.9% reported an improved understanding of safe birth control options.

## 4. Discussion

The current study is the first qualitative patient-centered evaluation of a combined COB clinic as a model for prenatal care. Despite the low respondent rate, pregnant individuals within this study reported high satisfaction with a team-based approach and improved knowledge of their heart disease as a result of dual counseling. However, participants did not perceive the same increase in understanding of safe and effective contraception despite targeted counseling. As the cardio-obstetrics paradigm is a relatively new area of medicine, there are minimal data to support the benefit of a combined clinic, especially those whose care delivery occurs during the same patient encounter. We have previously reported on the successful clinical outcomes in our combined clinic [7]. These new findings alongside our previously published data demonstrate that a combined care approach for high-risk pregnancies affected by cardiovascular disease is a patient-satisfier and improves both knowledge and clinical outcomes.

Participants in the COB clinic were overall satisfied with their care and the communication both between providers, and amongst providers and the patient. Caring for high-risk pregnant people can prove challenging as various types of specialists deliver medical management throughout gestation depending on the clinical disease status. This can lead to disjointed communication and both patient and provider frustration, as well as poor outcomes due to lack of clear patient handoffs. However, having multiple physicians in the same room during an exam can be intimidating, especially when sensitive health issues arise such as pregnancy complications. For the most part, women in the COB clinic did not perceive a team-based approach as intimidating and expressed a greater desire for this type of model as opposed to separate visits. In fact, patients reported such high satisfaction that they would recommend the program to family or friends and utilize the same services in a subsequent pregnancy. Previous studies in other areas of medicine have shown that patients receiving care in this type of similar model are more likely to be adherent to recommendations and perceive their providers’ communication as more streamline and clear [10,15]. Indeed, women in the COB clinic had a greater understanding of their heart disease and the risks to any future pregnancy as a result of combined counseling. Traditional patient dissatisfaction centers around communication failure in the doctor-patient relationship. This combined model addresses these shortcomings by having all necessary care providers within the same space, virtually eliminating communication breakdown. As well, it was encouraged to have a family member or friend attend the appointment (pre-COVID) to foster an atmosphere of shared decision making and enhanced communication; this was the exact perception reported by the patients. Lastly, multiple visits to a doctor or hospital involve multiple co-pays and facility fees, both of which are eliminated within a combined model as both cardiac and pregnancy needs were addressed during one outpatient appointment and likely added to the enhanced satisfaction scores.

Heart disease poses multiple challenges for family planning as estrogen-containing hormones are contraindicated in many types of heart disease including hypertension, coronary artery disease, or a history of myocardial infarction [16]. Many women are unaware of the dangers of exogenous estrogens and either avoid contraception altogether or use methods that are less effective or contraindicated for their disease state. As over 50% of all pregnancies are unplanned, this can pose increased risk for adverse obstetrical and cardiovascular outcomes in women with underlying cardiac disease. Familiarity with the different types of available contraceptive options, their risk and safety profiles, efficacy rates, and any contraindications to use, particularly in cardiac disease states is imperative and is the shared responsibility of all providers who care for reproductive-aged people [16]. Many participants in the COB clinic were not utilizing contraception prior to presentation. Of those who were, the most common form utilized were condoms, which have an inherently high failure rate. A critical part of the COB clinic counseling includes contraceptive management and safe utilization, which is outlined in our standardized obstetrical plan of care. Contraceptive options are usually discussed during the third trimester or at the postpartum visit. However, following participation in the clinic, many women did not feel they had a good grasp of safe and effective contraceptive options, which is different than what has previously been reported in the literature for postpartum patients with CV disease [17]. This is likely due to one of several reasons: (1) presenting to the COB clinic earlier in gestation with no further COB visits (i.e., consultation only) and therefore not receiving contraceptive counseling or (2) not presenting for postpartum care. Our findings bolsters the need for all care providers—not just obstetricians—to take the time at each and every patient encounter to not only discuss options but assess understanding at follow-up visits. Only 17.7% of participants were utilizing long-acting contraception, or LARCs, which are highly efficacious with a minimal side effect profile compared to other forms. These forms of contraception are the preferred modality for women with heart disease and should be discussed in COB clinics by cardiologist and obstetricians alike [16,18].

There are several limitations of this study which warrant discussion. Although the clinic has cared for over 1500 women during the timeframe, only 900 had provided an email of whom 119 were able to be reached and agreed to participate. The majority of study participants received their total obstetrical care exclusively by the MFM team, as compared to those who did not respond. Those who did respond were more likely to have their medical care provided by a physician within the Saint Luke’s Health System (i.e., established cardiology patient prior to pregnancy). The overall breakdown of cardiac disease in the participants did not differ significantly from those who did not respond. However, the majority of our participants had private insurance and did not report barriers to care, which is likely different to the non-respondent group (as ~45% of all pregnant patients evaluated in the MFM clinic on are on public assistance (i.e., Medicaid)) [19,20].

Response rates to health care questionnaires have been declining over the last decade and our attempt at reaching this high risk group of individuals was no exception [14,21,22]. The reason for such a low response rate is likely multifactorial. People tend to respond to surveys if they are shorter and digitized; although we simplified this process by sending a link, it may have been too long and led to survey fatigue. Moreover, Missouri has many rural communities and obstetrical care deserts where internet access is limited, and the majority of patients evaluated in the COB clinic were from rural zip codes outside the Kansas City metropolitan area. The research team deliberately chose not to contact patients by phone as contact from the providers in the clinic could have biased the satisfaction responses. Limiting contact to email likely contributed to lower response rates [9]. It is also possible that those patients who were not able to be reached or who deliberately chose not to participate had lower satisfaction or additional social or economic barriers, again leading to inclusion or ascertainment bias [23]. It is well documented that non-responders more often have underlying medical co-morbidities such as depression, cognitive limitations or substance abuse disorders [24]. In a state where the leading cause for maternal mortality for white women is mental health disorders (including substance abuse), it was an oversight by not asking this specific question and ensuring representation of this group of people. Lastly, the study was performed during the COVID pandemic and given the multitude of reported stressors experienced during this time frame (i.e., job loss, financial difficulties, etc.) for many families, responding to a questionnaire may not have been top priority [19]. Finally, post-implementation questionnaires can lead to recall bias, which is likely as many of these women were participants in the COB clinic up to 4 years prior to being contacted for the study. Given the low response rate, our findings may not be generalizable to other COB clinics.

Despite the low response rate, there are many strengths and future quality improvement opportunities gained from these data. To our knowledge, this is the first study assessing patient perceptions and experiences in a cardio-obstetrics clinic in a cohort of pregnant individuals with cardiovascular disease. It was important to note from a quality improvement standpoint, the majority of patients were referred by their general obstetricians. Although this facilitates care during pregnancy, those women with known cardiac disease planning a future pregnancy are not being reached in the primary care setting. As well, women with congenital or structural disease are often managed by pediatric cardiologist until 21 and are not yet of the age where gynecology care is recommended. Therefore, there is an opportunity for the COB clinicians to collaborate with both primary care and pediatric groups to encourage contraceptive use for young women with underlying cardiac disease and potentially avoid unplanned pregnancies.

Recently, a joint presidential advisory from the American Heart Association and ACOG reiterated the importance of coordinated health care delivery between cardiologists and obstetricians to allow for better assessment of patient needs and improve outcomes during pregnancy and postpartum timeframes [5,6]. There is an ever-growing need for the development of COB clinics and collaboration within this space. Current data support improved outcomes within other areas of medicine where a multidisciplinary approach is implemented to manage complex conditions such diabetes (cardio-metabolic clinics), cancer (cardio-oncology) and neurologic conditions [8,25], and heart disease in pregnancy is no different. Multidisciplinary collaboration has led to decreased overall mortality in critical non-obstetric cardiac patients and given that cardiac disease is the number one cause of maternal death, it only makes sense to introduce these types of models into the COB paradigm [26]. Studies have shown that these multidisciplinary models not only improve outcomes, they are patient satisfiers, enhance patient knowledge, and increase patient-perceived improvement in outcomes as compared to traditional care models [10,12]. In addition to positive patient outcomes, combined care clinics have been shown to increase provider satisfaction and reduce provider burnout [27,28]. However, there are few published data specific to the impact of COB teams on pregnancy outcomes and overall satisfaction in pregnant persons with cardiovascular disease. There is no published work addressing the degree of benefit achieved by implementation of COB teams, as there is no comparison to standard of care. Multicenter studies are needed to provide sufficient power to study differences in outcomes between multidisciplinary team approaches and usual care and to provide comparisons between different algorithms that result in optimal outcomes.

## 5. Conclusions

Cardiovascular disease is the newly recognized leading cause of maternal death and continues to rise with each published report from state maternal mortality review committees [4,29]. Management to minimize maternal mortality and morbidity requires input from multiple providers including cardiologists with expertise in the hemodynamic effects and pharmacotherapeutics of pregnancy, maternal fetal medicine experts comfortable in managing high-risk patients, anesthesiologists trained in obstetrics care, and highly skilled nursing staff. Collaborative care models in other areas of medicine have proven beneficial for both the patient and the provider and there is a need to tailor similar models in the cardio-obstetrics space. Previous studies have pointed towards improved outcomes for women with heart disease during gestation but this is the first study recognizing that this benefit extends beyond quantitative outcomes [7,13]. Pregnant patients with cardiac disease receiving care in a combined COB clinic report high levels of satisfaction, perceived enhanced provider-to-provider and provider-to-patient communication, and increased knowledge about their own cardiac disease as a result of being part of these types of clinics. More objective metrics for knowledge acquisition following contraceptive counseling, such as patient-reported postpartum contractive use, is an opportunity for future work.

## Figures and Tables

**Table 1:** Demographic and Sociodemographic Data for 119 Women in the Combined Cardio-Obstetrics Clinic at Saint Luke’s Hospital of Kansas City, MO.

Characteristic	*n* (%) or Mean ± SD
**Age**	30.9 ± 7.5
**Parity**	1.6 ± 5.2
**Race**	
American Indian	5 (4.2)
Asian/Pacific Islander	3 (2.5)
Black	12 (10.1)
Hispanic	2 (1.7)
White	101 (84.9)
Other	3 (2.5)
**Patients with more than one pregnancy as part of HDPP**	28 (24.3)
**Has a significant other or support person involved with the pregnancy**	107 (89.9)
**Marital status**	
Married	88 (74.6)
Single	22 (18.6)
Divorced/separated	7 (5.9)
Registered partnership	1 (0.8)
**Level of education**	
Less than High School	2 (1.7)
High school	12 (10.2)
Some college	38 (32.2)
Completed college	40 (33.9)
Graduate or higher	26 (22.0)
**Employed during pregnancy**	84 (71.2)
**Type of insurance**	
Medicaid	15 (12.8)
Private	89 (76.1)
Self-pay	4 (3.4)
Government	9 (7.7)
**Type of Cardiac Disease**	
Congenital	32 (26.9)
Arrhythmia	29 (24.4)
Acquired	18 (15.1)
Other	40 (33.6)
**Has previously had to choose between Rx medications and paying for groceries**	11 (9.4)
**Has previously had to choose between Rx medications and paying rent**	12 (10.3)
**Chose not to attend clinic visit due to:**	
Transportation barriers to accessing healthcare	5 (4.2)
Could not afford co-pays for clinic visits	3 (2.5)
Childcare as barrier to accessing healthcare	4 (3.4)
Did not believe it would make a difference	0 (0)
Fear of discrimination from the medical system/provider	0 (0)
Fear of being told to have an abortion	0 (0)
Fear of being made to feel bad about decision to become pregnant/continue pregnancy	0 (0)
Significant stress from other life events (e.g., Emotional, relationship, work)	0 (0)

Legend: OCP—oral contraceptive, IUD—intrauterine device, Rx—prescription, COB—cardio-obstetrics.

**Table 2:** Self-reported birth control use prior to index pregnancy.

Type of Contraception Use Prior to Pregnancy *	*n* (%)
Combined OCP	17 (14.3)
IUD	17 (14.3)
Depo-Provera/Nexplanon	4 (3.4)
Vaginal ring	4 (3.4)
Condom	18 (15.1)
Natural family planning	13 (10.9)
Progesterone only pill	16 (13.4)
Spermicide	2 (1.7)
Withdrawal	7 (5.9)
Contraceptive patch	1 (0.8)
Other	3 (2.5)
None	43 (36.1)

* some women were utilizing multiple methods at one time.

**Table 3:** Care patterns and preferences of women within the combined Cardio-Obstetrics clinic of Saint Luke’s Hospital of Kansas City.

Referral Pattern and Patient Preference Questions	Positive Number of Patient Responses *n* (%)
Appointment with cardiology prior to visit	84 (71.8)
Appointment with high-risk OB prior to visit	57 (48.3)
Intimidated by multiple providers in same exam room	17 (14.3)
Would have preferred to see providers at separate visits	9 (7.6)
Perceived improvement of communication with multiple providers in same exam room	102 (86.4)
Perceived improvement in overall care with multiple providers in same exam room	103 (86.6)
Attend postpartum visit	100 (84.7)
Would use program for subsequent pregnancies	84 (70.6)
Would recommend program to family or friends	106 (89.1)
Do you feel that a combined visit saved you money	87 (73.1)
Had support person attend COB visit	65 (54.6)
Support person present enhanced understanding	60 (95.2)

**Table 4:** Patient satisfaction with combined Cardio-Obstetrics clinic of Saint Luke’s Hospital of Kansas City, based on scale of 1–7.

Satisfaction Parameter	Total Cohort Score (Mean ± SD) *n* = 119
Quality of care received	6.2 ± 1.5
Seeing OB and cardiologist in same visit	6.3 ± 1.5
Communication amongst team members	6.1 ± 1.6
Communication regarding care and expectations	6.1 ± 1.5
Delivery location	
Communication between clinic doctors and delivery team at Saint Luke’s main campus	6.0 ± 1.7
Communication between clinic doctors and delivery team at other hospitals	6.1 ± 1.6

Satisfaction scores for all 6 questions were similar within subgroups (race, education, marital status, and insurance) (Appendix B).

**Table 5:** Perceived knowledge gained as a result of participating in the combined Cardio-Obstetrics clinic of Saint Luke’s Hospital of Kansas City.

Knowledge Prior to Program	Positive Response *n* (%)	Perceived Knowledge Acquisition	Positive Response *n* (%)
Cardiology counseling about effects of pregnancy on heart condition	41 (35.0)	As a result of being part of the program, knowledge gained about pregnancy impacts on heart disease	88 (73.9)
Counseling about effects of heart medications during pregnancy	36 (30.5)		
Counseled against pregnancy due to heart condition	13 (11)		
Counseled about safe birth control options	50 (42.4)	Improved understanding of safe birth control options	66 (55.9)

## Data Availability

Not applicable.

## Appendix A. Patient Questionnaire

**Please Answer the Following 6 Questions Using a Likert Satisfaction Scale with 7 Being Extremely Satisfied and 1 Very Unsatisfied**									
**Question**		**Very Unsatisfied**	**Unsatisfied**	**Somewhat Unsatisfied**	**Somewhat Satisfied**	**Satisfied**	**Very Satisfied**	**Extremely Satisfied (i.e., Best Medical Experience)**	**Not Applicable**
1.	How satisfied were you with the quality of care you received from the Saint Luke’s HDPP?	1	2	3	4	5	6	7	N/A
2.	How satisfied were you with being able to see a high risk OB provider and cardiologist in one visit?	1	2	3	4	5	6	7	N/A
3.	How satisfied were you with the communication between the team members (cardiologist, OB provider, nurses, etc.) caring for you?	1	2	3	4	5	6	7	N/A
4.	How satisfied were you with our communication with you regarding your pregnancy care and what to expect?	1	2	3	4	5	6	7	N/A
5.	If you delivered at Saint Luke’s Hospital Plaza campus, how satisfied were you with the hospital team’s communication with you about your plan of care, symptoms, and what to expect while hospitalized?	1	2	3	4	5	6	7	N/A
6.	If you delivered at a location other than Saint Luke’s Hospital Plaza campus, how satisfied were you with the hospital team’s communication with you about your plan of care, symptoms, and what to expect while hospitalized?	1	2	3	4	5	6	7	N/A

**QUESTION**	**CHECK ALL THAT APPLY**							
7. How did you hear about the cardiac-OB clinic	OB provider		Cardiologist	Friend or Family	Marketing or advertising campaign	Social Media	Other	
8. What type of birth control were you most recently using (the year before) prior to the pregnancy for which you had care in our program?	None		Depo-Provera Shot	Nexplanon	Condoms	IUD	Natural family planning	
	Combo pills		Vaginal Ring	Progesterone only pills	Spermicide	Withdrawal	Contraceptive patch	
	Other							
9. Did you ever not attend one of your visits during pregnancy due to any of the following?	Not enough money for co-pay	Not enough money for transportation		Lack of access to transportation	Did not believe it would make a difference in my pregnancy	Fear of discrimination from the medical system/provider		Fear they would tell me to have an abortion
	Fear of being made to feel bad about my decision to become pregnant/continue pregnancy			Did not have childcare for other children	Significant stress from other life events (e.g., Emotional, relationship, work)	Other		

**QUESTION**	**YES**	**NO**
10. Would you use this program for a future pregnancy?		
11. Would you Recommend this program to a friend or family member?		
12. As a result of the program, do you have a better understanding about how pregnancy impacts your heart disease?		
13. As a result of the combined clinic structure, do you feel like you saved money by avoiding separate visits?		
14. As a result of the program, do you have a better understanding about what are the safest options of birth control for your type of heart disease?		
15. Was your significant other involved with the pregnancy?		
16. Did anyone attend your heart disease in pregnancy appointments with you?		
17. If yes, was it helpful to have a person present at your appointments?		
18. Was it intimidating or concerning that a group of physicians came into your exam room at the same time?		
19. Do you think having your doctors all in the same room improved communication during your pregnancy?		
20. Do you think having your doctors in the same room improved the care of your pregnancy?		
21. Would you have preferred to see a cardiologist separate from the high-risk pregnancy doctor?		
22. Did you attend your postpartum visit (sometime before 6 weeks after delivery)?		
23. Prior to your visit in the Heart Disease in Pregnancy Clinic, had you seen a cardiologist?		
24. If you were under the care of a cardiologist prior to pregnancy, did they counsel you about the effects of pregnancy on your heart condition?		
25. Did you have an appointment with a high risk OB prior to your visit in the Heart Disease in Pregnancy Program?		
26. Did any care provider tell you that you should not become pregnant because of your heart condition?		
27. Before pregnancy, had any provider counseled you about the effects of your heart condition on your pregnancy?		
28. Before pregnancy, had any provider counseled you about the effects of medications needed to treat your heart condition and the effects on pregnancy?		
29. Before pregnancy, had any of your care providers (example: family practice, regular OB, pediatrician, etc.) spoken with you about birth control options that are safe for your condition?		
30. Did you hold a job outside the home when you were pregnant?		
31. Did your pregnancy or any events associated with your pregnancy cause you to reconsider or stop working?		

## Appendix B. Satisfaction Scores Stratified by Race, Reported in Means ± SD

**Question**	**Race**			***p*-Value**
	**White** ***n* = 96**	**Black** ***n* = 11**	**Other** ***n* = 12**	
32. Satisfied with the quality of care you received from COB?	6.2 ± 1.5	5.9 ± 1.9	6.3 ± 1.1	0.691
33. Satisfied with seeing high risk OB and cardiologist in 1 visit?	6.3 ± 1.5	6.9 ± 0.3	5.6 ± 2.0	0.171
34. Satisfied with the comm between the team members?	6.1 ± 1.5	5.9 ± 2.0	5.9 ± 1.5	0.669
35. Satisfied with our comm with you regarding your care?	6.2 ± 1.4	5.5 ± 2.2	5.7 ± 1.7	0.277
36. Satisfied with the hospital communication about your plan of care (main campus)?	6.1 ± 1.6	5.5 ± 2.4	6.3 ± 1.2	0.685
37. Satisfied with the hospital comm about your plan of care (not main campus)?	6.2 ± 1.4	6.0 ± 1.4	5.1 ± 2.5	0.618
